# MicroRNA-1298 is downregulated in non-small cell lung cancer and suppresses tumor progression in tumor cells

**DOI:** 10.1186/s13000-019-0911-4

**Published:** 2019-12-04

**Authors:** Zhonghai Du, Jun Wu, Juan Wang, Yan Liang, Sensen Zhang, Zhimei Shang, Wenchao Zuo

**Affiliations:** Cancer center, Weifang Traditional Chinese Hospital, No. 1055 Weizhou Road, Weifang, 261041 Shandong China

**Keywords:** MicroRNA-1298, Non-small lung cancer; prognosis; proliferation, Migration, Invasion

## Abstract

**Background:**

MicroRNAs (miRNAs) have been reported to serve pivotal roles in tumorigenesis. This study sough to assess the expression and clinical significance of microRNA-1298 (miR-1298) in patients with non-small cell lung cancer (NSCLC), and explore the functional role of miR-1298 in tumorigenesis.

**Methods:**

One hundred and twenty-one NSCLC patients were recruited in this study. The expression of miR-1298 was estimated using quantitative real-time PCR. Kaplan-Meier survival curves and Cox regression analysis were used to evaluate the prognostic value of miR-1298. Gain- and loss-of-function experiments were preformed to explore the biological function of miR-1298 in NSCLC cells.

**Results:**

Expression levels of miR-1298 were downregulated in NSCLC tissues and cells compared with the corresponding normal controls. The decreased expression of miR-1298 was associated with patients’ lymph node metastasis and TNM stage. The low expression of miR-1298 predicted poor overall survival and served as an independent prognostic indicator in NSCLC patients. According to the cell experiments, NSCLC cell proliferation, migration and invasion were inhibited by the overexpression of miR-1298.

**Conclusion:**

All the data indicated that the downregulation of miR-1298 predicts poor prognosis of NSCLC, and the overexpression of miR-1298 in NSCLC cells leads to inhibited tumorigenesis. The aberrant miR-1298 may serve as a novel biomarker and therapeutic target in NSCLC.

## Introduction

Lung cancer is one of the most serious global health burden, which ranks the number one cause of cancer morbidity and mortality worldwide [1]. It is classified into two major subtypes, including small cell lung cancer (SCLC) and non-small cell lung cancer (NSCLC). NSCLC is a common type and accounts for appropriately 80% of all lung cancer cases [2]. Patients with early stage of NSCLC have no obvious typical clinical manifestation, leading to that most of cancer patients are initially diagnosed with advanced tumors [3]. Previous studies indicate that the occurrence of NSCLC can be promoted by some risk factors, among them the smoking represents a significantly influential one [4]. Currently, some therapeutic strategies, such as surgery, chemotherapy and radiotherapy, have been improved and applied in the treatment of lung cancer, but the prognosis of NSCLC patients remains quite dismal [5]. Thus, reliable predictors for the clinical outcomes of patients are necessary to improve the prognosis of NSCLC.

It is known that tumorigenesis is a complex process with abundant deregulated genes [6]. MicroRNAs (miRNAs) are considered a group of important molecules during tumorigenesis [7]. MiRNAs are small noncoding RNAs with important regulatory role in gene expression at the post-transcriptional level [8]. The functional miRNAs can be involved in various cellular processes, such as cell viability, migration, invasion, differentiation, cell cycle and cell apoptosis [9]. It is demonstrated that the biological function of miRNAs is generally achieved by regulating target genes by binding the 3′-untranslated region (3′-UTR) of the target messenger RNA (mRNA) [10]. Accumulated evidence shown that the miRNAs can be involved in the regulation of tumor cell biological processes by targeting oncogenes or tumor suppressors [11–13]. Therefore, to identify functional miRNAs that abnormally expressed in NSCLC may provide novel therapeutic ideas for cancer treatment. A study by Xu et al. analyzed the deregulated miRNAs in NSCLC in silico, and microRNA-1298 (miR-1298) was found to be decreased in tumor cases compared with the normal controls [14]. Another study by Zhou et al. indicates that miR-1298 is closely related with the KRAS mutation, which is determined as a key event during lung cancer progression [15]. However, the expression patterns of miR-1298 in NSCLC patients and its biological function are still unclear.

To further improve the prognosis of NSCLC and understand the role of miR-1298 in this malignancy, this study sought to assess the expression of miR-1298 in NSCLC tissues and cell lines, evaluate its prognostic performance and explore the biological function of miR-1298 in tumor progression of NSCLC.

## Materials and methods

### Patients

A total of 121 patients, who were pathologically diagnosed with NSCLC in cancer center of Weifang Traditional Chinese Hospital between 2010 and 2013, were included in this study. None of them had received any preoperative anti-tumor therapy. During the surgery in Weifang Traditional Chinese Hospital, 121 paired tumor tissues and adjacent normal tissues were collected and immediately frozen in liquid nitrogen for further use. This study recorded the demographic and clinicopathological characteristics of the patients, and enrolled all the patients in a 5-year follow-up survival survey to obtain the data for subsequent analyses. The experimental procedures were approved by the Ethic Committee of Weifang Traditional Chinese Hospital, and a written informed consent was provided by each participant.

### Cell culture and transfection

Four NSCLC cell lines (A549, H1299, PC9 and NCIH460) and one normal human airway epithelial cell line (16HBE) were obtained from the Cell Bank of Chinese Academy of Science (Shanghai, China). All the cells were cultured in Roswell Park Memorial Institute 1640 (RPMI 1640; Gibco, Carlsbad, CA) medium added with 10% fetal bovine serum (FBS; Gibco, Carlsbad, CA) in an incubator under an atmosphere containing 5% CO_2_ at 37 °C. miR-1298 mimic, miR-1298 inhibitor and miRNA negative control sequence (miR-NC) were synthesized at GenePharma (Shanghai, China) and used for in vitro regulation of miR-1298. The cell transfection was performed using Lipofectamine 3000 (Invitrogen, Carlsbad, CA, USA) as per the manufacturer’s instructions. Subsequent cell experiments were conducted at 48 h post-transfection.

### Quantitative real-time PCR (qRT-PCR)

Total RNA in tissues and cells was extracted using Trizol reagent (Invitrogen, Carlsbad, CA, USA), then cDNA was synthesized from the RNA by a PrimeScript RT reagent kit (Takara Bio, Shiga, Japan) following the manufacturer’s directions. The PCR amplification was carried out using a SYBR Green I Master Mix kit (Invitrogen, Carlsbad, CA, USA) and a 7500 Real-Time PCR System (Applied Biosystems, USA). The final relative expression of miR-1298 was calculated using the 2^−ΔΔCt^ method and normalized to U6.

### Cell proliferation assay

Cell counting kit-8 (CCK-8; Dojindo, Kumamoto, Japan) was used to estimate the cell proliferation of NSCLC cells. The cells with a density of 3000 cells/well were seeded into 96-well plates and incubated for 0, 24, 48 and 72 h, followed by treatment with CCK-8 solution for 4 h. After the incubation, a microplate reader (Bio-Tek, Winooski, VT, USA) was used to measure the absorbance at 450 nm.

### Cell migration and invasion assay

Cell migration and invasion were evaluated using the Transwell chambers (Corning, NY, USA). The chambers used for invasion assay were coated with Matrigel, but those for migration assay had no need for coating. Cells (density of 2 × 10^5^ cell/well) were seeded into the upper chambers with serum - free medium, then cultured at 37 °C. The lower chambers were filled with medium supplemented with 10% FBS as chemoattractant. After 24 h of incubation, the cells moved to the lower chambers were fixed with methanol and stained with crystal violet. The cells in random five fields were counted under a light microscope.

### Statistical analysis

The data from all the analyses were expressed as mean ± SD and analyzed using SPSS 21.0 (SPSS Inc., Chicago, IL, USA) and GraphPad Prism 7.0 (GraphPad Software, Inc., USA). Student’s t test and one-way ANOVA were used for comparison between groups. Chi-square test was applied to evaluate the relationship between miR-1298 expression and the clinicopathological features of NSCLC patients. Survival curves were plotted using a Kaplan-Meier method and compared with the log-rank test. Cox regression analysis to confirm the independency of miR-1298 as a prognostic predictor. A *P* value of less than 0.05 was considered statistically significant.

## Results

### Downregulated expression of miR-1298 in NSCLC

**Fig. 1:**
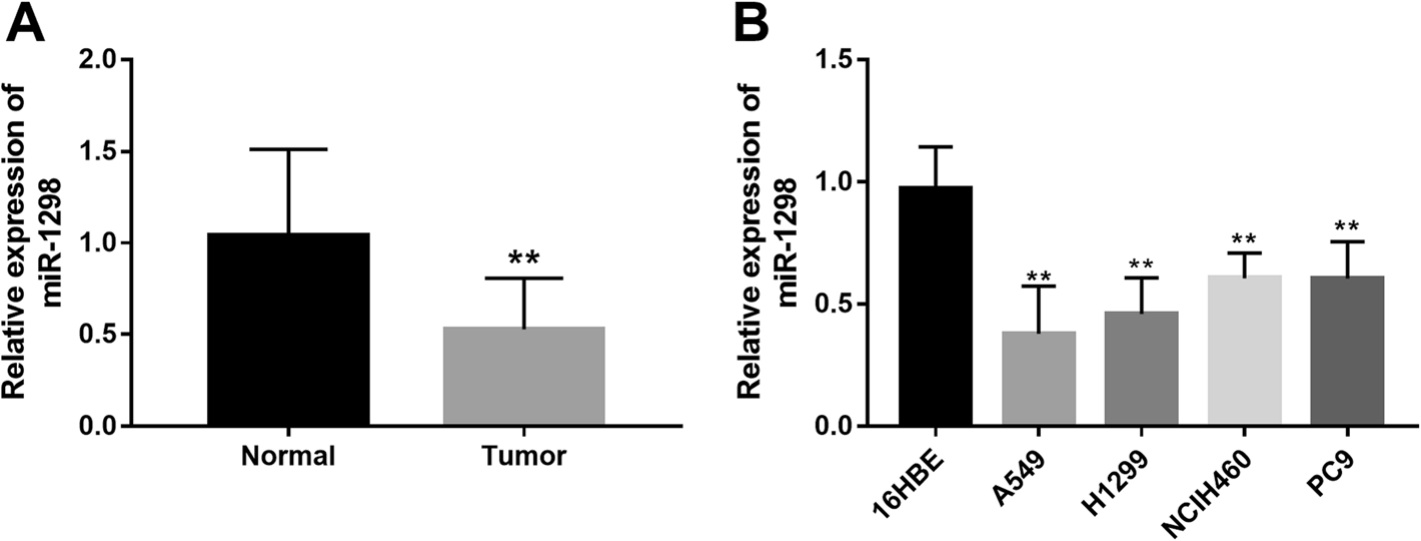
Decreased expression of miR-1298 in NSCLC tissues and cell lines. A. Expression of miR-1298 in NSCLC tumor tissues was lower than that in the normal tissues. B. Expression of miR-1298 was downregulated in the four NSCLC cell lines (A549, H1299, NCIH460 and PC9) compared with the normal cell line (16HBE). **P < 0.01

The qRT-PCR results shown in Fig. 1a indicated that the expression of miR-1298 was significantly decreased in NSCLC tissues compared with the adjacent normal tissues (*P* < 0.01). Similarly, in comparison with the normal cell line 16HBE, the expression of miR-1298 was found to be downregulated in the four NSCLC cell lines (all *P* < 0.01, Fig. 1b).

### Association between miR-1298 expression and the clinicopathological data of NSCLC patients

The demographic and clinicopathological characteristics of the patients were listed in Table 1, including age, gender, smoking history, tumor size, lymph node metastasis and TNM stage. To facilitate the subsequent analyses, the expression of miR-1298 was divided into low expression group and high expression group according to the mean expression value (0.527). By the relationship analysis between miR-1298 and these features, we found that the expression of miR-1298 was associated with patients’ lymph node metastasis (*P* = 0.024) and TNM stage (*P* = 0.009). No significant relationship was obtained between miR-1298 and the remaining features (*P* > 0.05 for age, gender, smoking history and tumor size).

**Table 1:** Association of miR-1298 and the clinical characteristics of the NSCLC patients

Features	Total No. *N* = 121	miR-99b expression		*P* values
		Low (*n* = 62)	High (*n* = 59)	
Age (years)				0.564
≤ 60	38	18	20	
> 60	83	44	39	
Gender				0.864
Female	44	23	21	
Male	77	39	38	
Smoking history				0.837
No	44	22	22	
Yes	77	40	37	
Tumor size (cm)				0.218
≤ 3	69	32	37	
> 3	52	30	22	
Lymph node metastasis				0.024
Negative	57	23	34	
Positive	64	39	25	
TNM stage				0.009
I - II	53	20	33	
III - IV	68	42	26	

### Decreased miR-1298 expression predicts poor prognosis in patients with NSCLC

**Fig. 2:**
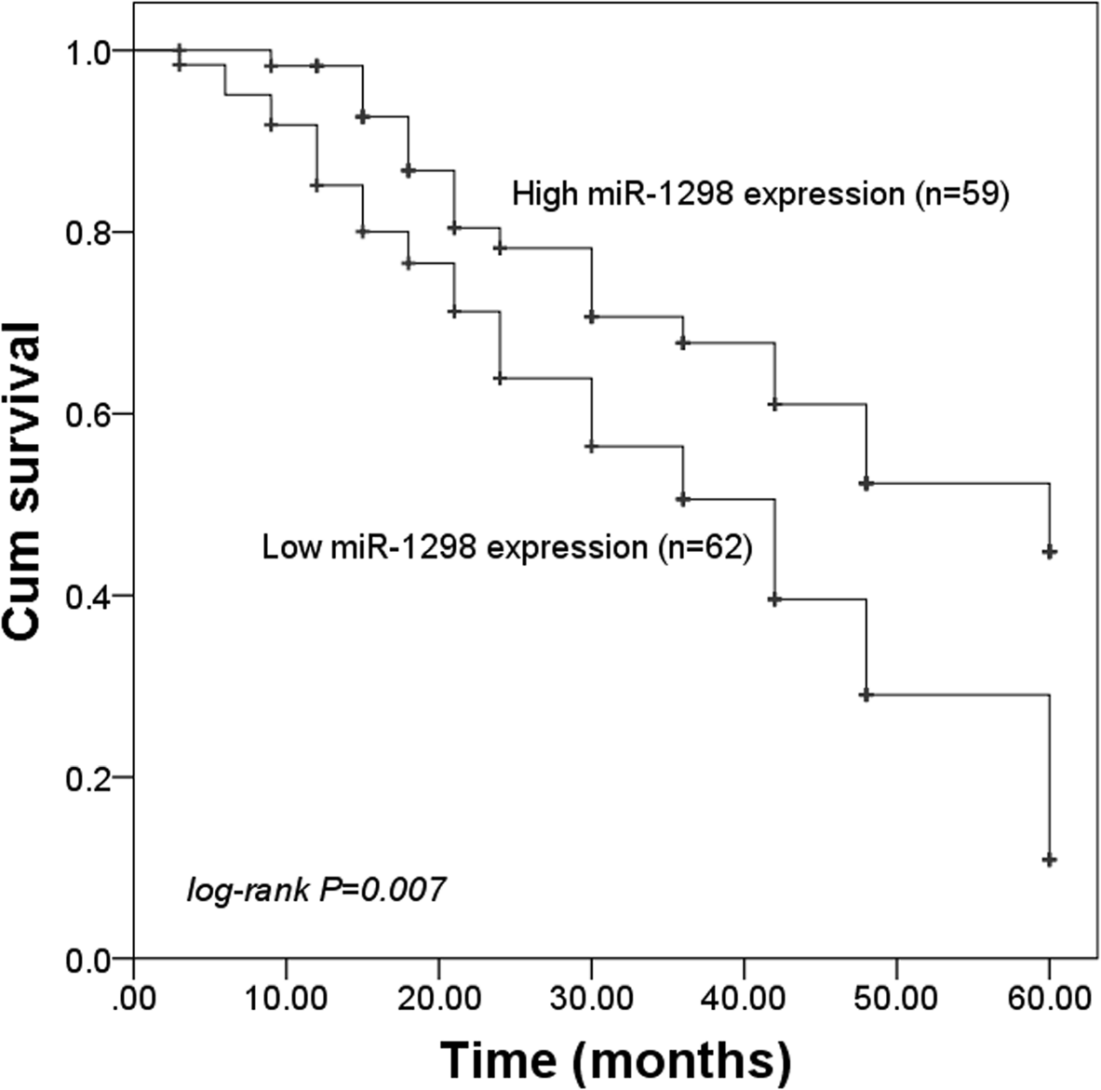
Kaplan-Meier survival curves for the patients with NSCLC. Low miR-1298 expression was associated with shorter survival time when compared with the high miR-1298 expression. Log-rank *P* = 0.007

This study recorded the survival information of the patients by a 5-year follow-up survey. The survival curves were plotted and shown in Fig. 2, which implied that the patients with low miR-1298 expression levels had a poor overall survival compared with those with high miR-1298 expression levels (log-rank *P* = 0.007). Furthermore, by considering the clinical features into a Cox regression analysis, we drew a conclusion that miR-1298 was an independent prognostic factor in the patients with NSCLC (HR = 1.805, 95% CI = 1.023–3.186, *P* = 0.042, Table 2).

**Table 2:** Cox regression analysis in the patients with NSCLC

Variables	Multivariate analysis		
	HR	95% CI	*P* value
miR-1298	1.805	1.023–3.186	0.042
Age	1.651	0.889–3.064	0.112
Gender	1.065	0.627–1.808	0.817
Smoking history	1.238	0.722–2.124	0.438
Tumor size	1.452	0.873–2.471	0.151
Lymph node metastasis	1.607	0.923–2.797	0.094
TNM stage	1.724	1.014–2.933	0.044

### Overexpression of miR-1298 inhibits NSCLC cell proliferation

**Fig. 3:**
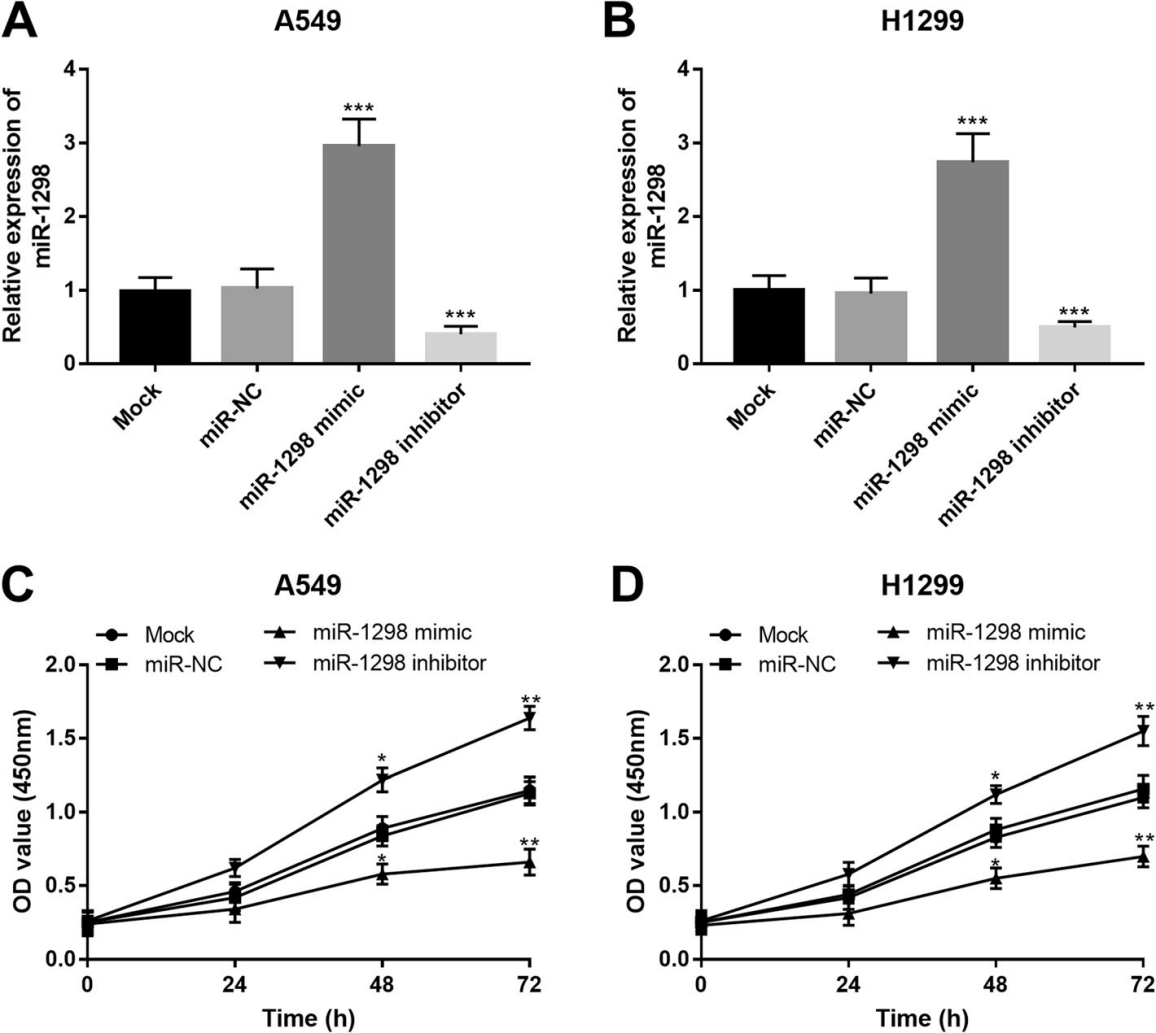
In vitro regulation of miR-1298 and its effect on NSCLC cell proliferation in A549 and H1299 cell lines. A and B. miR-1298 expression was promoted by the miR-1298 mimic, but was suppressed by the miR-1298 inhibitor. C and D. Cell proliferation was promoted by the downregulation of miR-1298, while was inhibited by the upregulation of miR-1298. *P < 0.05, **P < 0.01 ***P < 0.001

Given the dysregulation of miR-1298 in NSCLC tissues and cells, this study further explored its potential functional role in tumor progression by function-gain and -loss experiments. According to the cell transfection, miR-1298 expression was upregulated by the miR-1298 mimic, while was downregulated by the miR-1298 inhibitor in both A549 and H1299 (all *P* < 0.001, Fig. 3a and b). By CCK-8 assay, we observed that the overexpression of miR-1298 in NSCLC cells led to the inhibition in cell proliferation, whereas the knockdown of miR-1298 resulted in the promoted cell proliferation in both A549 and H1299 cells (all *P* < 0.05, Fig. 3c and d).

### Upregulation of miR-1298 suppresses cell migration and invasion in NSCLC cells

**Fig. 4:**
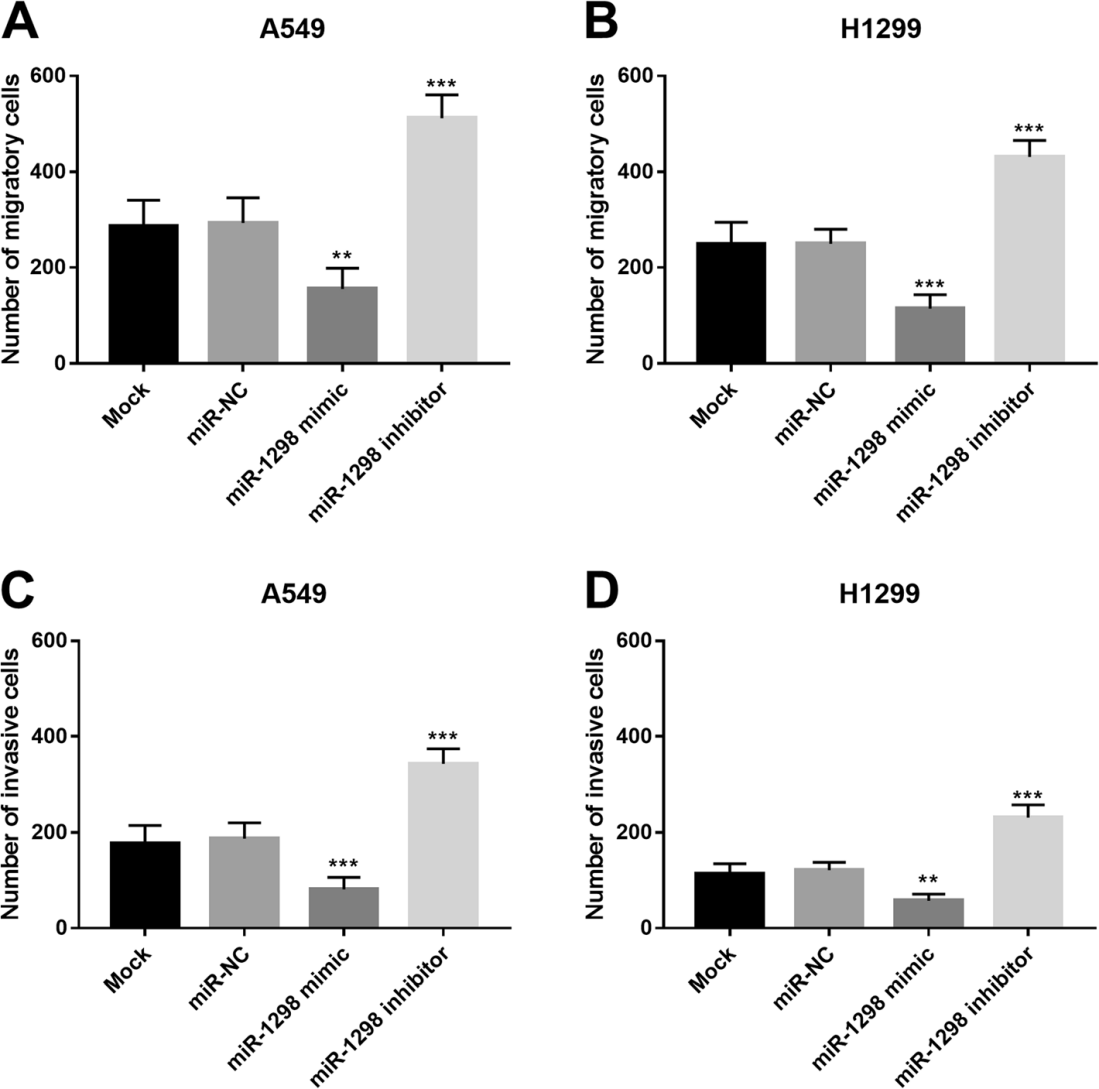
Effects of miR-1298 on NSCLC cell migration and invasion in A549 and H1299 cell lines. A and B. The overexpression of miR-1298 inhibited tumor cell migration, but the reduction of miR-1298 promoted tumor cell migration. C and D. Tumor cell invasion was facilitated by the knockdown of miR-1298, whereas was suppressed by the overexpression of miR-1298. **P < 0.01 ***P < 0.001

According to the Transwell assay, this study counted the migratory and invasive cells in the Transwell chambers. The results shown in Fig. 4a and b indicated that the cell migration of A549 and H1299 was suppressed by the overexpression of miR-1298, but was enhanced by the reduction of miR-1298 (all *P* < 0.01). Similarly, the upregulation of miR-1298 also inhibited the NSCLC cell invasion, while the downregulation of miR-1298 resulted in the opposite results (*P* < 0.01, Fig. 4c and d).

## Discussion

Lung cancer is a serious global malignancy, and early diagnosis and poor prognosis remain two challenges for the disease treatment. This study focused the role of miR-1298 in the prognosis and tumor progression of NSCLC. In NSCLC patients, we found that the expression of miR-1298 was significantly downregulated in tumor tissues compared with the normal controls, and its expression was related with patients’ lymph node metastasis and TNM stage. The survival analysis indicated that patients with low miR-1298 had a poor overall survival, and the expression of miR-1298 might serve as an independent prognostic indicator for the patients. The subsequent functional-gain and -loss cell experiments implied that the overexpression of miR-1298 could suppress NSCLC cell proliferation, migration and invasion, while the knockdown of miR-1298 led to the opposite results.

Accumulated studies highlight the important role of miRNAs in various human diseases, especially in human cancers [16, 17]. It is well known that the tumorigenesis is a complex process involving the dysregulation of abundant key molecules, including miRNAs [18, 19]. In lung cancer patients, a variety of aberrant miRNAs have been identified with pivotal roles in the tumor occurrence and development [20]. For example, Tian et al. found the upregulated miR-181b-5p and downregulated miR-486-5p in the serum samples of NSCLC patients, which might be involved in tumorigenesis by targeting tumor-related key genes [21]. Qin et al. gave evidence for decreased miR-340 expression in NSCLC patients and its important role in tumor development and tumor cell proliferation [22]. Tang et al. indicated that the expression of miR-650 was elevated in NSCLC tissues and cell lines and acted as a biomarker and therapeutic target in the pathogenesis of NSCLC [23]. In the present study, miR-1298 expression was detected to be downregulated expressed in NSCLC tissues and cell lines, and the significant association was found between low miR-1298 expression and positive patients’ lymph node metastasis and advanced TNM stage. These clinical analysis results indicated that miR-1298 might be involved in the tumor development of NSCLC. Consistent with our expression results, a previous study by Xu and colleagues also found the decreased miR-1298 expression in NSCLC by a bioinformatics analysis [14]. In addition to NSCLC, the reduced miR-1298 expression has also been identified in patients with gastric cancer [24] and neuroglioma [25], indicating the tumor suppressor role of miR-1298 in tumorigenesis.

Since most of NSCLC patients are diagnosed with advanced tumors initially, their overall survival outcomes remain dismal. Currently, miRNAs have been considered a group of efficient biomarkers to predicate the prognosis of various human malignancies. Upregulated miR-4709 expression in colon adenocarcinoma predicts a poor prognosis in cancer patients [26]. Downregulation of miR-29c-3p is associated with the poor overall survival in patients with laryngeal squamous cell carcinoma [27]. Overexpression of miR-193b in glioma patients serves as a candidate diagnostic and prognostic biomarker [28]. In NSCLC patients, there are also some miRNAs members that have been determined as potential prognostic indicators, such as miR-126 [29], miR-451 [30] and miR-421 [31]. According to the survival analysis, this study obtained evidence for miR-1298 as an independent prognostic biomarker in the patients with NSCLC, which indicated by the survival curves and Cox regression analysis data. Thus, we believed that the decreased miR-1298 expression might have a potential to predict the poor prognosis of NSCLC.

Emerging studies have focused on the regulatory effects of miR-1298 on tumor cell proliferation, migration and invasion in some human malignancies [24, 32]. In gastric cancer, Qiu et al. demonstrated that miR-1298 overexpression could inhibit tumor cell proliferation and invasion [24]. In bladder cancer, Li et al. draw a conclusion that miR-1298 serves a tumor suppressor by suppressing tumor cell proliferation, migration and invasion [32]. In the tumor progression of NSCLC, some aberrant miRNAs have important functional roles by regulating tumor cell processes, and have been determined as potential therapeutic targets [33–35]. Considering the dysregulation of miR-1298 in NSCLC, this study further explored its biological function in tumor progression. By in vitro regulation of miR-1298, the NSCLC cell proliferation, migration and invasion were inhibited by the overexpression of miR-1298, while were facilitated by the knockdown of miR-1298, indicating the tumor suppressor role of miR-1298 in NSCLC progression.

Although this study provided a novel insight into the functional role of miR-1298 in NSCLC, the underlying mechanisms remain unclear. A study by Zhou et al. indicated that miR-1298 could inhibit the mutation of KRAS, which serves as an oncogene in human malignancies, leading to the suppression in tumor growth [15]. In addition, Li et al. demonstrated that miR-1298-5p exerted inhibiting effects on bladder cancer cell proliferation and invasion by downregulating connexin 43 [32]. In the tumorigenesis of NSCLC, whether the molecules mentioned above were also involved in the mechanisms underlying the role of miR-1298 is uncertain, which warrant further investigations.

In conclusion, the data of this study give evidence for the downregulated expression of miR-1298 in NSCLC tissues as a candidate prognostic biomarker, and demonstrated that miR-1298 acts as a tumor suppressor in NSCLC progression by inhibiting tumor cell proliferation, migration and invasion. Thus, the methods to increase miR-1298 expression may be novel therapeutic strategies for the treatment of NSCLC.

## Data Availability

All data generated or analyzed during this study are included in this article.

## Abbreviations

3′-UTR: 3′-untranslated region
CCK-8: Cell counting kit-8
miR-1298: microRNA-1298
miRNAs: microRNAs
miR-NC: miRNA negative control sequence
NSCLC: non-small cell lung cancer
qRT-PCR: quantitative Real-time PCR
RPMI 1640: Roswell Park Memorial Institute 1640
SCLC: small cell lung cancer
